# Bioprocessing of *Sargassum fusiforme* via *Lactobacillus* Fermentation: Effects on Nutrient Composition, Organoleptic Properties, and In Vitro Antioxidant and Hypoglycemic Activities

**DOI:** 10.3390/foods14081385

**Published:** 2025-04-17

**Authors:** Chao Zhang, Houyun Zhang, Shengli Lin, Laijin Su

**Affiliations:** 1 College of Life and Environmental Science, Wenzhou University, Wenzhou 325035, China; zhangchao6823@163.com (C.Z.);; 2 Zhejiang Provincial Key Laboratory for Water Environment and Marine Biological Resources Protection, Wenzhou University, Wenzhou 325035, China; 3 Wenzhou Academy of Agricultural Science, Wenzhou Characteristic Food Resources Engineering and Technology Research Center, Wenzhou 325006, China

**Keywords:** *Sargassum fusiforme*, probiotic fermentation, active constituents, volatile components, antioxidant and hypoglycemic activities

## Abstract

*Sargassum fusiforme* is an abundant source of biologically active compounds that are released during fermentation. However, the effects of *Lactobacillus* fermentation on the nutrient composition of *S. fusiforme* have yet to be sufficiently determined. In this study, we used five strains of *Lactobacillus* to ferment *S. fusiforme* and examined changes in the bioactive components, volatile compounds, and bioactivities of the fermentation supernatants. Among the assessed strains, fermentation with *Lactobacillus delbrueckii* promoted significant increases in the total phenolic contents, and fermentation with all strains contributed to reductions in the levels of undesirable volatile compounds associated with the characteristic odor of *S. fusiforme*. In addition, *S. fusiforme* fermented using *L. delbrueckii* showed superior ABTS radical scavenging activity, whereas *S. fusiforme* fermented using *L. plantarum* FY03 (PF-3) or *L. plantarum* FY02 (PF-2) showed enhanced DPPH radical scavenging capacity, and fermentation using *L. rhamnosus* promoted the highest ferric-ion-reducing power. Moreover, the inhibition of α-glucosidase activity increased by 2.0- to 3.0-fold in fermented *S. fusiforme*, whereas the inhibition of α-amylase activity was only significantly augmented by the PF-2 and PF-3 strains. These findings highlight the potential health benefits of *Lactobacillus*-fermented *S. fusiforme*, particularly the enhanced antioxidant activities and the capacity to inhibit α-glucosidase and α-amylase activities.

## 1. Introduction

Recently, there has been growing interest in functional foods. In this context, *Sargassum fusiforme* has become known as a viable source of bioactive chemicals with antioxidant and hypoglycemic properties. *Sargassum fusiforme* is a perennial brown alga belonging to the genus Sargassum (family Sargassaceae). The thallus is erect, highly branched, and yellowish–brown in color. This species is predominantly distributed in China, Japan, and the Korean Peninsula [1]. This seaweed is rich in biologically active components, including *Sargassum fusiforme* fucoidan, sargassuol A, sargassuol B, dehydrololiolide, calcium, iron, and magnesium. Notably, it exhibits antioxidant, hypoglycemic, immune-modulating, and anti-inflammatory activities [2,3,4,5]. “Shen Nong’s Canon of Materia Medica” documents that *Sargassum fusiforme* is effective in curing goiter, facilitating urination, and alleviating edema [1]. However, the currently marketed products of *S. fusiforme* tend to be limited to minimally processed commodities, and, notably, the commonly employed processing methods, such as natural drying, hot air drying, ultraviolet radiation, and high-temperature treatments, have been reported to cause significant nutrient loss, thereby contributing to a deterioration in product quality [6,7]. Moreover, the strong fishy odor associated with these primary processed products tends to limit consumer acceptance, thereby posing a challenge to the sustainable development of the industry. In addition, a further hurdle to effective processing is the impedance of the cell wall on the release of nutrients and bioactive compounds from *S. fusiforme* cells [8,9]. Consequently, there is an imperative to develop innovative processing technologies that can contribute to enhancing the nutritional value and bioactive potential of *S. fusiforme* products, whilst also facilitating the diversification of product types.

Fermentation, a time-honored technique for food processing and preservation, facilitates the release of bioactive compounds, whilst also contributing to the augmentation of flavor profiles and nutritional quality, and extending the shelf life of products by inhibiting microbial spoilage [10]. Accumulating evidence indicates that a regular intake of fermented dairy and vegetable products is correlated with measurable health benefits. Specifically, these foods may contribute to strengthening immune responses, reducing the risk of disease, modulating the composition of the gut microbiota, and promoting systemic antioxidant activity [11]. Bioactive peptides and polyamines produced by lactic acid bacteria during food fermentation exhibit beneficial effects on immunometabolic regulation and cardiovascular health [12]. Furthermore, SCFAs and lactates generated by lactic acid bacteria enhance gut health by influencing the makeup of the gut microbiota and maintaining the integrity of the gut barrier [13].

Given their functional and safety-related properties, lactic acid bacteria (LAB) are utilized extensively in the food industry [14]. For example, by suppressing competing microbiota, including spoilage organisms, via the secretion of metabolites such as organic acids, these bacteria can contribute to extending the shelf life of food [15]. Notably, the lactobacillin-mediated fermentation of red algae has been established to enhance the bioactivity of the resulting products, including the inhibition of pancreatic lipase and increases in antimicrobial efficacy [16]. We accordingly speculate that adopting this approach for the processing of *S. fusiforme* could contribute to an enhancement of its nutritional quality, whilst reducing the characteristic undesirable odors.

The findings of previous studies have revealed the hypoglycemic properties of extracts obtained from *S. fusiforme*; however, although in theory, LAB fermentation would similarly contribute to enhancing the bioactivity and flavor profiles of seaweeds, there has to date been comparatively little empirical research assessing the fermentative processing of *S. fusiforme*. Accordingly, to establish whether LAB-mediated fermentation would contribute to further enhancements in hypoglycemic efficacy and other beneficial properties, we systematically analyzed bioactive changes during the fermentation of *S. fusiforme* using five selected *Lactobacillus* strains (*Lactobacillus plantarum* FY02, *L. plantarum* FY03, *L. rhamnosus* GJ01, *L. acidophilus*, and *L. delbrueckii*), based on the quantification of changes in bioactive components and assessments of in vitro antioxidant capacity and hypoglycemic activity using standardized assays. Our findings in this regard provide valuable insights for the development of value-added *S. fusiforme* products with enhanced organoleptic properties and health benefits, which are considered key drivers of industrial upscaling in the seaweed sector.

## 2. Materials and Methods

### 2.1. Materials

Fresh samples of *S. fusiforme* were obtained from Dongtou, Wenzhou, Zhejiang Province, China. *L. plantarum* FY02, *L. plantarum* FY03, and *L. rhamnosus* were isolated in our laboratory, and *L. acidophilus* and *L. delbrueckii* were obtained from the Guangdong Provincial Microbial Strain Preservation (Guangzhou, China). α-Glucosidase and α-amylase were acquired from Shanghai McLean Biochemical Science and Technology Co. (Shanghai, China).

### 2.2. Preparation of S. fusiforme Fermentation Supernatants

LAB strains (*L. plantarum* FY02, *L. plantarum* FY03, *L. rhamnosus*, *L. acidophilus*, and *L. delbrueckii*) were individually inoculated into MRS culture medium and incubated at 37 °C for 24 h. Thereafter, having centrifuged the resulting cultures for 5 min, the supernatants were discarded and the pelleted cells were rinsed with saline and subsequently resuspended in sterile saline to an OD_600_ of 1.00 ± 0.01.

Having initially cleaned fresh samples of *S. fusiforme*, aliquots of 10 g *S. fusiforme* were mixed with distilled water at a 1:10 (*w*/*v*) ratio (final volume: 100 mL) and homogenized using a JYL-Y92 high-speed food processor (Jiuyang Co., Ltd., Jinan, China) under the following conditions: 20,000 rpm for 3 cycles (30 s each cycle, 10 s interval) at 25 °C.

The homogenized slurries were transferred into 250 mL conical flasks and sterilized via autoclaving at 121 °C for 15 min, and then cooled to 25 °C. The initial pH was measured as 5.58 ± 0.02.

Each bacterial strain (*Lactobacillus* spp.) was inoculated into sterilized *S. fusiforme* slurry at 4% (*v*/*v*). Fermentation was performed in an SP-50B biochemical incubator (Beijing Hengnuo Li Xingke Technology Co., Ltd., Beijing, China) at 37 °C for 24 h under static anaerobic conditions. For the non-fermented control group, 4% (*v*/*v*) sterile physiological saline (0.9% NaCl) was added to the *S. fusiforme* slurry instead of bacterial inoculum to ensure equivalent liquid volumes.

After fermentation, *S. fusiforme* slurries were centrifuged to separate supernatants. The supernatants were stored at −80 °C until analysis.

The sterilized *S. fusiforme* (pH 5.58 ± 0.02) was then separately inoculated with suspensions of each of the five *Lactobacillus* strains at a 4% (*v*/*v*) ratio and fermented for 24 h at 37 °C in an SP-50B biochemical incubator (Beijing Hengnuo Li Xingke Technology Co., Ltd., Beijing, China). Thereafter, the culture supernatants were collected via centrifugation. As a non-fermented control, we cultured uninoculated *S. fusiforme* under the same conditions.

### 2.3. Assessment of pH Levels and Total Acidity

The pH of the cultures was measured directly using a pH meter, and the total acidity of samples was determined via acid–base titration using NaOH (0.01 mol/L), with phenolphthalein used as an indicator. The total acid concentration is expressed in g/L.

### 2.4. Determination of Total Phenolic Contents

The phenolic contents of fermentation supernatants were determined using the procedure described by Habschied et al. [17]. Absorbance was measured at 760 nm, with concentrations being calculated from a standard curve prepared using gallic acid. The concentration is expressed in mg/mL.

### 2.5. Determination of Total Flavonoid Contents

The total flavonoid contents of supernatants were determined using the method described by Zhao et al. [18]. Briefly, supernatant samples (0.5 mL) were mixed with NaNO_2_ solution (0.3 mL, 5%), followed by the addition of solutions of Al(NO_3_)_3_ (0.3 mL, 10%) and NaOH (4 mL, 4%). Having made the volumes of sample up to 10 mL with 70% ethanol, absorbance was recorded at 510 nm. The concentration is expressed in mg/mL.

### 2.6. Determination of Short-Chain Fatty Acids

Concentrations of short-chain fatty acids (SCFAs) in supernatants were analyzed using the procedure described by Zang et al. [19]. Briefly, to 100 μL samples of supernatants, we added 50 μL of 75% sulfuric acid and 10 μL of internal standard, followed by vortex mixing. Thereafter, having added 140 μL of ether, the samples were centrifuged at 14,000 rpm for 15 min. The analysis was performed using liquid chromatography–mass spectrometry. The concentrations are expressed in μg/mL.

The chromatographic conditions: an HPFFAP capillary column (30 m × 0.25 mm × 0.25 μm; Agilent J&W Scientific, Folsom, CA, USA); high-purity helium (purity not less than 99.999%) as the carrier gas at a flow rate of 1.0 mL/min; an inlet port temperature of 230 °C; and an injection volume of 1 µL. The sample was injected via shunt with a shunt ratio of 10:1 and a solvent delay of 2.5 min. The initial temperature of the column chamber was 100 °C, and the temperature was programmed to increase to 160 °C at 15 °C/min and hold for 1 min, then increase to 200 °C at 40 °C/min and hold for 1 min, and then hold for 1 min at 220 °C after the run.

The mass spectrometry conditions: an electron bombardment ion source, an ion source temperature of 230 °C, a quadrupole temperature of 150 °C, a transmission line temperature of 230 °C, and an electron energy of 70 eV. The ion scanning mode (SIM) was selected.

### 2.7. Examination of the Volatile Components of S. fusiforme Supernatants

The methodology used for the analysis of supernatant volatile components has been described previously by Yan et al. [20]. Briefly, 1 μL samples were introduced into 20 mL headspace injection vials and prepared for gas chromatography–ion mobility spectrometry (GC-IMS), with an incubation temperature of 50 °C, a rotational speed of 250 rpm, and an incubation time of 20 min. The injection volume was 0.5 mL.

The GC conditions: an MXT-WAX column (15 m, 0.53 mm, 1.0 μm df); a column temperature of 60 °C; and a carrier gas flow rate of 2 mL/min for 0–2 min, increasing to 10 mL/min within 5 min, 100 mL/min within 20 min, and 150 mL/min within 25 min.

The IMS conditions: high purity N_2_ (99.99%) as the drift gas; drift gas flow rates of 2, 10, and 150 mL/min for 0–2 min, 20 min, and 25 min, respectively; and a drift tube temperature of 45 °C.

### 2.8. Assessment of the Antioxidant Activity of Fermented S. fusiforme

#### 2.8.1. 2,2-Diphenyl-1-picrylhydrazyl Radical Scavenging

The rate of 2,2-diphenyl-1-picrylhydrazyl (DPPH) radical scavenging was assessed using the methodology outlined by Zhang et al., with minor modifications [21]. Samples (100 μL) were mixed with 100 μL of a 0.2 mmol/L DPPH solution in anhydrous ethanol, and the reaction was left to proceed for 30 min in a light-protected environment, with absorbances subsequently being recorded at 517 nm. Clearance rates are expressed in %.

#### 2.8.2. 2,2′-Azino-bis(3-ethylbenzothiazoline-6-sulfonic Acid Free Radical Scavenging

The rate of 2,2′-azino-bis(3-ethylbenzothiazoline-6-sulfonic acid (ABTS) free radical scavenging was assessed using the procedure described by Yang et al. [22]. Briefly, 200 μL of ABTS working solution was mixed with 10 μL of each sample, and following thorough mixing, the samples were allowed to stand for 6 min, after which absorbances were measured at 734 nm. Clearance rates are expressed in %.

#### 2.8.3. Ferric Ion-Reducing Antioxidant Power

The method used for determining the ferric ion-reducing antioxidant power (FRAP) of fermentation supernatants has previously been described by Ferrera et al. [23]. Briefly, 0.2 mL of sample was mixed with 3 mL of FRAP reagent and incubated in a water bath at 37 °C in for 30 min, and following this, absorbances were recorded at 593 nm. The ferric ion reduction capacity of the sample is expressed as the Fe^2+^ mmol/L required to achieve the same absorbance value.

### 2.9. Enzyme Inhibition Assays

#### 2.9.1. α-Amylase

The inhibition of α-amylase activity was assessed using the methodology established by Liu et al. [24]. Samples (0.25 mL) were initially mixed with 0.25 mL of 1 mg/mL α-amylase solution, with the subsequent addition of 0.5 mL of a 1.5% soluble starch solution following an initial 10 min incubation in a water bath at 37 °C for 5 min. Thereafter, having added 1 mL of DNS solution, the samples were heated in a boiling water bath for 5 min, and after allowing to cool to room temperature, the absorbances of samples were measured at 540 nm. The inhibition rate is expressed in %.

#### 2.9.2. α-Glucosidase

The inhibition of α-glucosidase activity was assessed using the method established by Wang et al. [25]. Briefly, each sample (25 μL) was added to 50 μL of 1.5 mmol/L p-nitrophenyl-α-D-glucopyranoside solution, and the samples were incubated at 37 °C for 10 min. Thirty microliters of 0.2 U/mL α-glucosidase solution was added, the samples were mixed, and the absorbance was measured at 405 nm after incubation at 37 °C. The inhibition rate is expressed in %.

### 2.10. Statistical Analysis

The data are presented as the means ± standard deviation. Analyses were performed with a one-way analysis of variance using SPSS 27 software (IBM, Armonk, NY, USA), with values deemed statistically significant at the *p* < 0.05 level.

## 3. Results and Discussion

### 3.1. Changes in Lactobacillus Fermentation Based on the pH and Total Acid Content of S. fusiforme Supernatants

**Figure 1:**
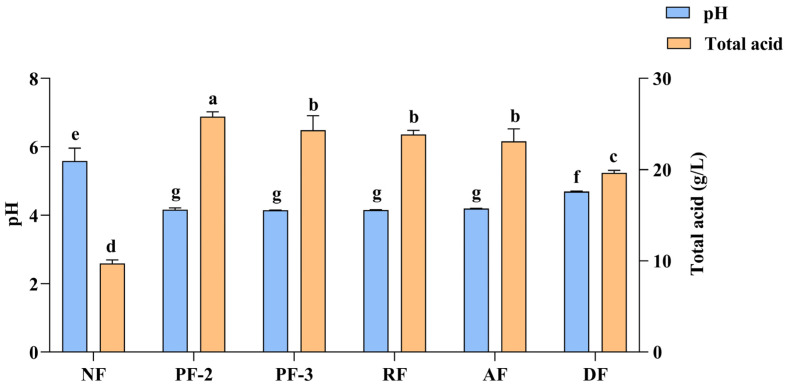
Effects of fermentation on the pH value and total acidity of fermentation supernatants of *Sargassum fusiforme*. NF, non-fermented supernatant of *Sargassum fusiforme*; PF-2, *Lactobacillus plantarum* FY02-fermented supernatant of *S. fusiforme*; PF-3, *L. plantarum* FY03-fermented supernatant of *S. fusiforme*; RF, *L. rhamnosus* GJ01-fermented supernatant of *S. fusiforme*; AF, *L. acidophilus*-fermented supernatant of *S. fusiforme*; and DF, *L. delbrueckii*-fermented supernatant of *S. fusiforme*. Different lowercase letters above bars denote significant differences between treatments (*p* < 0.05).

During the fermentation process, LAB can utilize the carbohydrates present in *S. fusiforme* as an energy source, thereby facilitating their growth and the production of metabolites. In this regard, pH and total acid contents can serve as key indicators of the extent of fermentation. LAB have been established to produce substantial quantities of organic acids during the fermentation process, including lactic acid, the concentrations of which can have a direct bearing on the shelf life and flavor of fermented products [26,27]. Consequently, the reduction in pH and the elevation in total acid concentration in the supernatant of *S. fusiforme* after fermentation, as evidenced in this study, can be ascribed predominantly to the accumulation of organic acids. As shown in Figure 1, compared with *S. fusiforme* cultured in the absence of *Lactobacillus*, there were statistically significant reductions in the pH and corresponding elevations in the total acid contents in *S. fusiforme* cultures following fermentation with each of the five assessed *Lactobacillus* strains (*p* < 0.05). Compared with the non-fermented *S. fusiforme*, we recorded increases of 2.0- to 2.7-fold in the total acid concentrations in the fermentation cultures, which is consistent with the observations documented by Kim et al. regarding the dynamics of pH during the *Lactobacillus*-mediated fermentation of *Actinidia deliciosa* [28]. Accordingly, the changes in the culture pH and total acidity observed in this study provide evidence to indicate that lactobacilli can utilize the carbohydrates in *S. fusiforme* for their growth and metabolic processes.

### 3.2. Effects of Lactobacillus Fermentation on the Total Phenolic Contents of S. fusiforme Fermentation Supernatants

**Figure 2:**
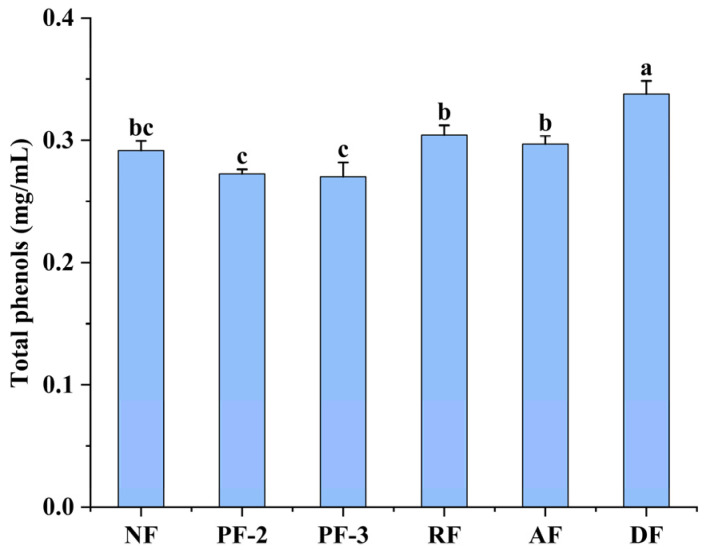
Total phenolic contents in fermentation supernatants of *Sargassum fusiforme*. NF, non-fermented supernatant of *S. fusiforme*; PF-2, *Lactobacillus plantarum* FY02-fermented supernatant of *S. fusiforme*; PF-3, *L. plantarum* FY03-fermented supernatant of *S. fusiforme*; RF, *L. rhamnosus* GJ01-fermented supernatant of *S. fusiforme*; AF, *L. acidophilus*-fermented supernatant of *S. fusiforme*; and DF, *L. delbrueckii*-fermented supernatant of *S. fusiforme*. Different lowercase letters above bars denote significant differences between treatments (*p* < 0.05).

Phenolic compounds are secondary plant metabolites that are often detected in abundance in fruits, vegetables, and grains, a number of which have been established to have anti-inflammatory, antioxidant, antitumor, anti-aging, anticarcinogenic, and antimicrobial properties [29]. The changes in the total phenolic contents preceding and following 24 h of *S. fusiforme* fermentation with different strains of *Lactobacillus* are illustrated in Figure 2. Compared with the non-fermented cultures, the total phenolic contents in *S. fusiforme* supernatants increased by 15.77% in the *L. delbrueckii* fermentation supernatants and declined by 7.03% and 7.94% in those of *L. plantarum* FY02 and FY03, respectively. Comparatively, with respect to the total phenolic contents, we detected no substantial differences between the non-fermented culture supernatant and the *L. rhamnosus-* and *L. acidophilus*-fermented supernatants (*p* > 0.05). We speculate that these differences could be attributable to differing degrees of utilization of *S. fusiforme* phenolics by the different species of *Lactobacillus*, as has been observed previously. For example, Li et al. detected a marked elevation in the total phenolic contents of the supernatant of *Bangia fuscopurpura* fermented with LAB compared those in a non-fermented supernatant [16]. Similarly, De Montijo-Prieto et al. reported substantial elevations in the total phenolics in response to fermentation with *Pediococcus pentosaceus* 4695T, *Lactobacillus brevis* 5354, *Pediococcus acidilactici* 5765T, and *L. plantarum* 9567, whereas in contrast, fermentation with *L. plantarum* 748T resulted in a reduction in the contents compared with the non-fermented control [30]. In summary, *Lactobacillus* species can have a substantial influence on the release and catabolism of polyphenolic substances in *S. fusiforme*.

### 3.3. Effects of Lactobacillus Fermentation on the Total Flavonoid Contents in S. fusiforme Fermentation Supernatants

**Figure 3:**
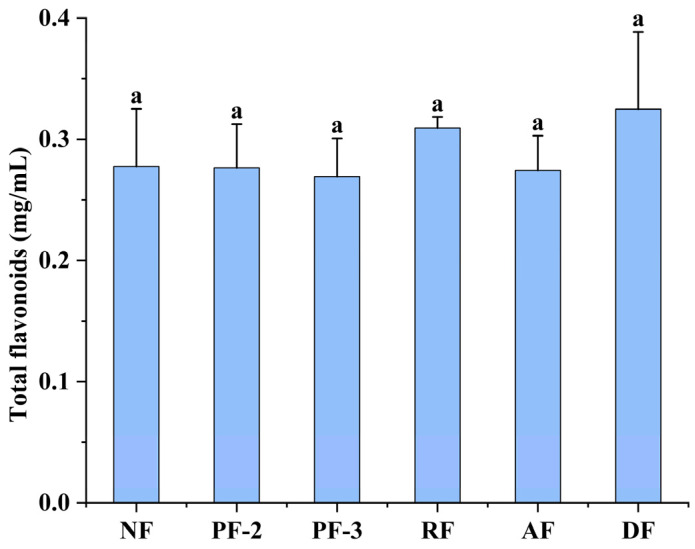
Total flavonoid content in fermentation supernatants of *Sargassum fusiforme*. NF, non-fermented supernatant of *S. fusiforme*; PF-2, *Lactobacillus plantarum* FY02-fermented supernatant of *S. fusiforme*; PF-3, *L. plantarum* FY03-fermented supernatant of *S. fusiforme*; RF, *L. rhamnosus* GJ01-fermented supernatant of *S. fusiforme*; AF, *L. acidophilus*-fermented supernatant of *S. fusiforme*; and DF, *L. delbrueckii*-fermented supernatant of *S. fusiforme*. Different lowercase letters above bars denote significant differences between treatments (*p* < 0.05).

Flavonoids are a class of secondary metabolites prevalent in a diverse range of plants, including fruits and vegetables, which have been established to have numerous bioactive properties, including antioxidant, anti-inflammatory, and anticancer activities [31]. As shown in Figure 3, there were no major changes in the total flavonoid contents of the supernatants obtained for fermented and non-fermented *S. fusiforme* (*p* > 0.05), which is consistent with the findings reported by Simsek et al., who observed no substantial change in the total flavonoid contents between fermented and non-fermented vegetable juices [32]. However, the authors of other studies have reported different findings. For example, Yao et al., who investigated changes in the flavonoid contents of fig juice fermented with *L. plantarum* strains SZ060 and SZ0606, detected significantly lower contents in juices subjected to mixed *L. plantarum* fermentation compared with the levels in the non-fermented juice. Conversely, the flavonoid contents in fig juice fermented with *L. plantarum* SZ0627 and naturally fermented fig juice were found to be significantly higher than those in non-fermented fig juice [33]. Given these observations, we accordingly speculate that our findings of no significant differences among the assessed fermentation groups with respect to total flavonoid contents may be attributed to the specificity of microorganisms for flavonoids in the fermentation system, and/or the nature of the fermentation substrate.

### 3.4. Analysis of Short-Chain Fatty Acids

The findings of previous studies have revealed that SCFAs play beneficial roles in the maintenance of intestinal and immunological homeostasis in humans [34]. The SCFAs analyzed in the present study are metabolites produced by LAB using carbohydrates and other substances in *S. fusiforme* for fermentation. As shown in Table 1, we identified eight fatty acids in the fermented *S. fusiforme* products examined in this study. Among these, we detected significant differences among the assessed *Lactobacillus* strains with respect to the contents of acetic acid (*p* < 0.05), with the highest concentrations being detected in *S. fusiforme* fermented with *L. delbrueckii*, quantified at 403.04 ± 3.38 μg/mL. This species similarly yielded the highest concentration of butyric acid in the fermentation products, at a level of 0.26 ± 0.01 μg/mL. Comparatively, the largest quantities of propionic acid were recorded in the fermentation products of the *L. rhamnosus* and *L. delbrueckii*, with levels of 0.74 ± 0.02 and 0.80 ± 0.01 μg/mL, respectively. These findings are comparable with those reported by Kang et al., who similarly detected differences in the contents of SCFAs produced by different LAB strains [35]. Collectively, these findings thus provide evidence indicating differences in the degree to which different *Lactobacillus* strains utilize fermentation substrates, as well as their inherent metabolic variability.

This study revealed that the analysis of the supernatant from fermented *S. fusiforme* indicated an increase in the overall concentration of short-chain fatty acids relative to the pre-fermentation phase. Research indicates that short-chain fatty acids in colonic contents predominantly consist of acetic, propionic, and butyric, with a molar ratio of approximately 60:20:20 in human colonic and fecal samples [36]. Consequently, the true impact of the SCFA ratio on gut health in this study requires examination through animal tests and clinical trials.

### 3.5. Effects of Lactobacillus Fermentation on the Volatile Constituents of S. fusiforme

**Figure 4:**
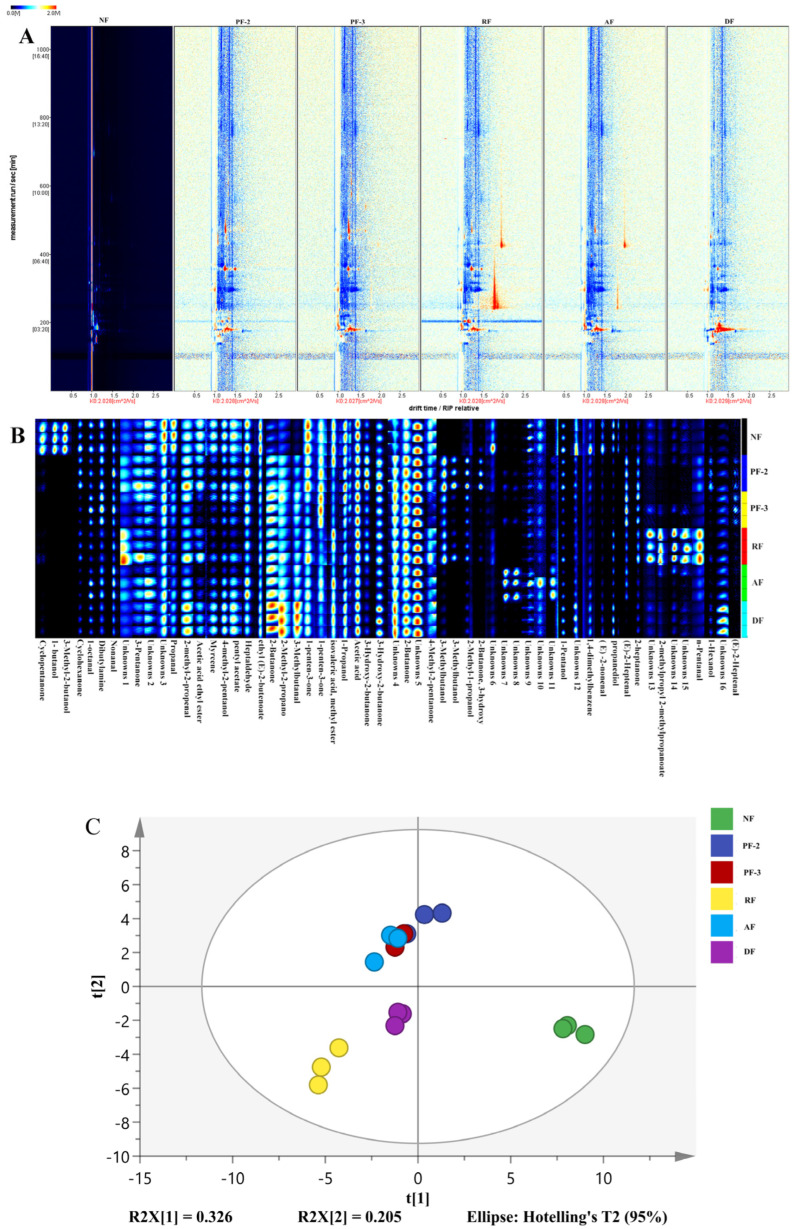
A two-dimensional map (**A**), chromatographic fingerprints (**B**), and principal component analysis (**C**) of the volatile compounds of *Sargassum fusiforme*. NF, non-fermented supernatant of *S. fusiforme*; PF-2, *Lactobacillus plantarum* FY02-fermented supernatant of *S. fusiforme*; PF-3, *L. plantarum* FY03-fermented supernatant of *S. fusiforme*; RF, *L. rhamnosus* GJ01-fermented supernatant of *S. fusiforme*; AF, *L. acidophilus*-fermented supernatant of *S. fusiforme*; and DF, *L. delbrueckii*-fermented supernatant of *S. fusiforme*.

As a source of food consumed by humans, the flavor profile of *S*. *fusiforme* is a key factor determining consumer acceptance. Although raw *S*. *fusiforme* products are characterized by a distinct fishy odor, microbial interactions with the volatile compounds inherent in the fermentation process can contribute to modifying their composition and sensory properties, thereby facilitating the elimination of this undesirable odor. In this study, we assessed the effects of *Lactobacillus* fermentation on the volatile components of *S. fusiforme* using GC-IMS, and our findings are presented in the two-dimensional spectra shown in Figure 4A. As a reference, we used a volatile matter spectrum obtained for non-fermented *S. fusiforme*, and the corresponding spectra for *S. fusiforme* fermented using the five assessed *Lactobacillus* strains were deduced from the reference to obtain comparative plots. The blue and red hues in these spectra signify that the contents of volatile components were, respectively, lower or higher than those in the non-fermented *S. fusiforme*.

Analyses of the volatile components in *S. fusiforme* revealed 53 distinct compounds among the six experimental groups (Table 2), comprising 8 ketones, 11 alcohols, 9 aldehydes, 5 esters, and 16 unidentified substances, along with minor quantities of benzene, amines, alkenes, and acids. To comparatively assess the effects of treatments on the volatile profiles, we analyzed chromatographic fingerprints, which thereby enabled us to visually differentiate these profiles based on concentration-dependent color gradients (Figure 4B), with brighter hues denoting higher concentrations.

With respect to the fishy odor of *S. fusiforme,* Chua et al. have established that this undesirable property is predominantly associated with the presence of aldehydes, ketones, and alcohols [37]. Our findings revealed that fermentation was characterized by significant reductions in the levels of 3-methyl-2-butanol, 1-butanol, and cyclopentanone, whereas in contrast, fermentation with *L. delbrueckii* was found to promote marked elevations in the concentrations of 2-butanone, 2-methyl-2-propanol, and 3-methylbutanal.

Notably, unidentified compounds 7–11 were found to show *L. acidophilus*-specific accumulation, whereas *L. rhamnosus*-fermented samples contained elevated levels of unknown compounds 13–15, 2-methylpropyl 2-methylpropanoate, and *n*-pentanal. We thus assume that these differential volatile patterns contribute the distinct organoleptic characteristics of the respective fermentation products.

To investigate the variability among the volatile compounds from *S. fusiforme* fermented with different lactobacilli, we performed principal component analysis (PCA), the results of which are presented in Figure 4C, indicating a clear pattern of segregation in the spatial distribution of volatile compounds from *S. fusiforme* in the different treatment groups. Samples from the non-fermented *S. fusiforme* show a distinct clustering in the fourth quadrant of the ordination plot, whereas those obtained from *S. fusiforme* fermented with *L. plantarum* FY02 mainly cluster in the first quadrant, and those obtained from *S. fusiforme* fermented with *L. plantarum* FY03 and *L. acidophilus* cluster in the second quadrant, thereby indicating that *S. fusiforme* fermented with the latter two strains had similar volatile compound profiles. Similar comparable profiles were indicated for *L. rhamnosus* and *L. delbrueckii* fermentation products, the samples of which co-cluster in the third quadrant.

In summary, the PCA plot clearly distinguishes the volatile components of *S. fusiforme* fermented with different *Lactobacillus* species, which may contribute to modifying the original fishy odor of this seaweed and thereby favorably enhance the organoleptic properties of these products.

### 3.6. Effects of Lactobacillus Fermentation of S. fusiforme on Antioxidant Activity

**Figure 5:**
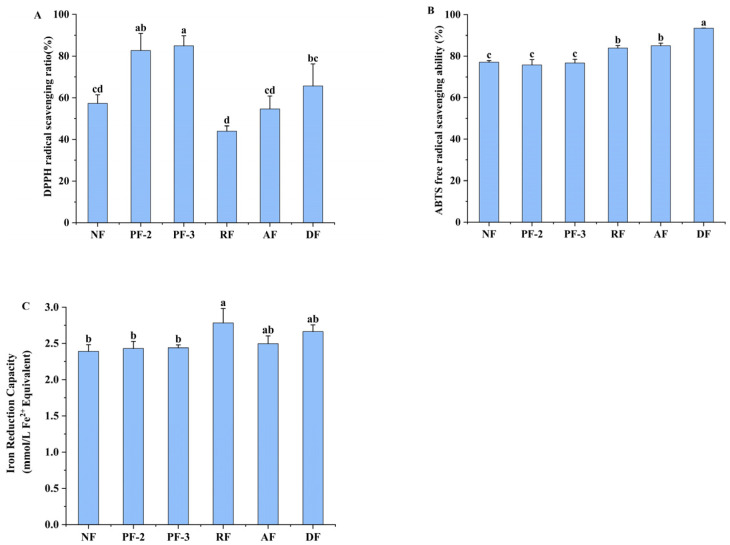
Rates of DPPH radical scavenging (**A**), rates of ABTS free radical scavenging (**B**), and ferric ion-reducing capacity (**C**) of fermentation supernatants of *Sargassum fusiforme*. NF, non-fermented supernatant of *S. fusiforme*; PF-2, *L. plantarum* FY02-fermented supernatant of *S. fusiforme*; PF-3, *L. plantarum* FY03-fermented supernatant of *S. fusiforme*; RF, *L. rhamnosus* GJ01-fermented supernatant of *S. fusiforme*; AF, *L. acidophilus*-fermented supernatant of *S. fusiforme*; and DF, *L. delbrueckii*-fermented supernatant of *S. fusiforme*. Different lowercase letters above bars denote significant differences between treatments (*p* < 0.05).

The in vitro antioxidant capacities of *S. fusiforme* fermented with different *Lactobacillus* strains were evaluated based on DPPH/ABTS radical scavenging assays and FRAP analysis. With respect to DPPH scavenging capacity, we detected significant inter-group variations (*p* < 0.05) (Figure 5A). Notably, compared with the non-fermentation treatment, fermentation with *L. plantarum* strains FY02 and FY03 was found to enhance the scavenging capacity by 44% and 48%, respectively, whereas statistically, compared with no fermentation, we detected little difference in the radical scavenging capacities in response to fermentation with *L. acidophilus* (*p* > 0.05). Contrastingly, *L. rhamnosus* fermentation reduced DPPH scavenging by 23% compared with that of the non-fermented *S. fusiforme*. These results are consistent with those obtained by Quan et al., who conducted a comparative study of six *Lactobacillus* strains used to ferment orange juice, and found that *L. plantarum*-fermented products were characterized by superior DPPH radical neutralization [38].

As shown in Figure 5B, ABTS free radical scavenging activity under fermentation with *L. rhamnosus*, *L. acidophilus*, and *L. delbrueckii* increased by 7%, 10%, and 20%, respectively, compared with that in the non-fermented *S. fusiforme*, whereas we detected no significant differences in activities between the *L. plantarum* FY02 and FY03 fermentations and non-fermentation (*p* > 0.05).

The ferric ion reduction capacities of *S. fusiforme* in response to the different treatments are shown in Figure 5C. Compared with the non-fermentation treatment, we recorded 16.43%, 4.48%, and 11.54% increases the ferric ion-reducing capacity of *S. fusiforme* fermented with *L. rhamnosus*, *L. acidophilus*, and *L. delbrueckii*, respectively, whereas again, we observed no significant differences in capacities between the *L. plantarum* FY02 and FY03 fermentations and non-fermentation (*p* > 0.05).

Collectively, these findings revealed that the fermentation of *S. fusiforme* with LAB influences its antioxidant activity, with notable differences being detected in the antioxidant capacities among *S. fusiforme* fermented with different *Lactobacillus* strains. We speculate that these differences may be associated with the capacity of different strains to catabolize certain antioxidant substances in *S. fusiforme*, as well as with the production of different metabolites by these bacteria.

### 3.7. Correlations Between Antioxidant Activity and Active Components in S. fusiforme

**Figure 6:**
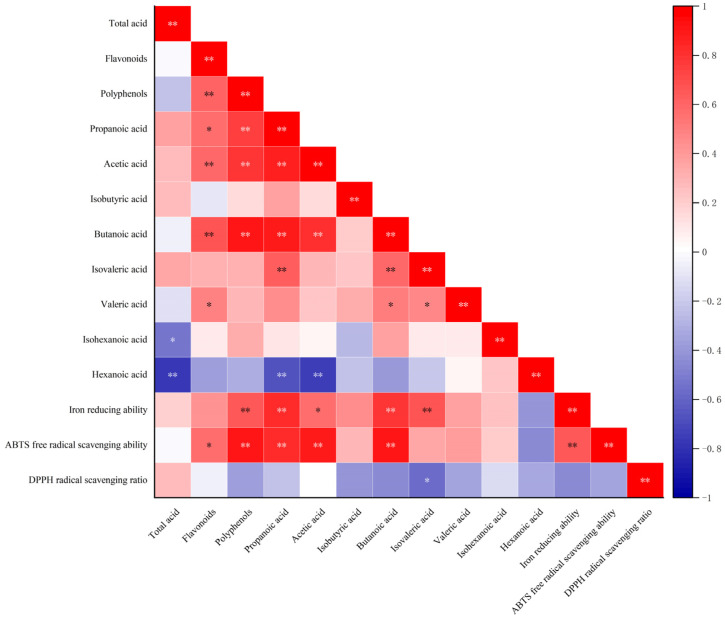
Analysis of correlations between antioxidant activity and active constituents in *Sargassum fusiforme*. *: *p* ≤ 0.05, **: *p* ≤ 0.01.

Certain bioactive components of *S. fusiform*, such as polyphenolic compounds, have been established to be closely associated with antioxidant activity. To gain further insights in this regard, we analyzed correlations between specific bioactive compounds in *S. fusiforme* and the antioxidant activities detected in fermentation products. The findings of previous studies that have examined LAB-fermented apple pulp have indicated that enhanced antioxidant activity may arise from increases in polyphenol content, particularly those of small-molecule forms [39]. As illustrated in Figure 6, we detected significant positive correlations between the ferric ion-reducing capacity and the levels of polyphenols and SFCAs (*p* < 0.05). Similarly, positive correlations were observed for the associations between ABTS radical scavenging capacity and the levels of flavonoids, polyphenols, and SFCAs (*p* < 0.05), whereas in contrast, we detected no significant associations between the DPPH radical scavenging capacity and polyphenol contents (*p* > 0.05), a finding that warrants further investigation. Collectively, these results indicated that LAB fermentation can augment the antioxidant potential of *S. fusiforme* by promoting the synthesis of polyphenols and other bioactive compounds.

### 3.8. Effects of Lactobacillus Fermentation on α-Glucosidase and α-Amylase Activities of S. fusiforme

Although dietary carbohydrates serve as an essential source of energy, the excessive consumption of carbohydrate-rich diets may lead to abnormal blood glucose levels. Accordingly, it is reasoned that impeding the rapid increase in postprandial blood glucose levels by controlling carbohydrate absorption would contribute to reducing the risk of heightened blood glucose levels [40]. Previous studies in this regard have shown that increased blood glucose concentrations are associated with the activities of carbohydrate-hydrolyzing enzymes, such as α-amylase and α-glucosidase [41], and thus, inhibiting the activities of these enzymes is considered an effective strategy for controlling rapid increases in blood glucose concentrations. A range of secondary metabolites in plants have been reported to have inhibitory activities against α-amylase and α-glucosidase [42]. Thus, given that in recent years, fermented foods have garnered heightened interest with respect to their unique flavor and enhanced biological activities [43], we sought to determine whether the LAB fermentation of *S. fusiforme* could contribute to augmenting the inhibition of α-amylase and α-glucosidase.

**Figure 7:**
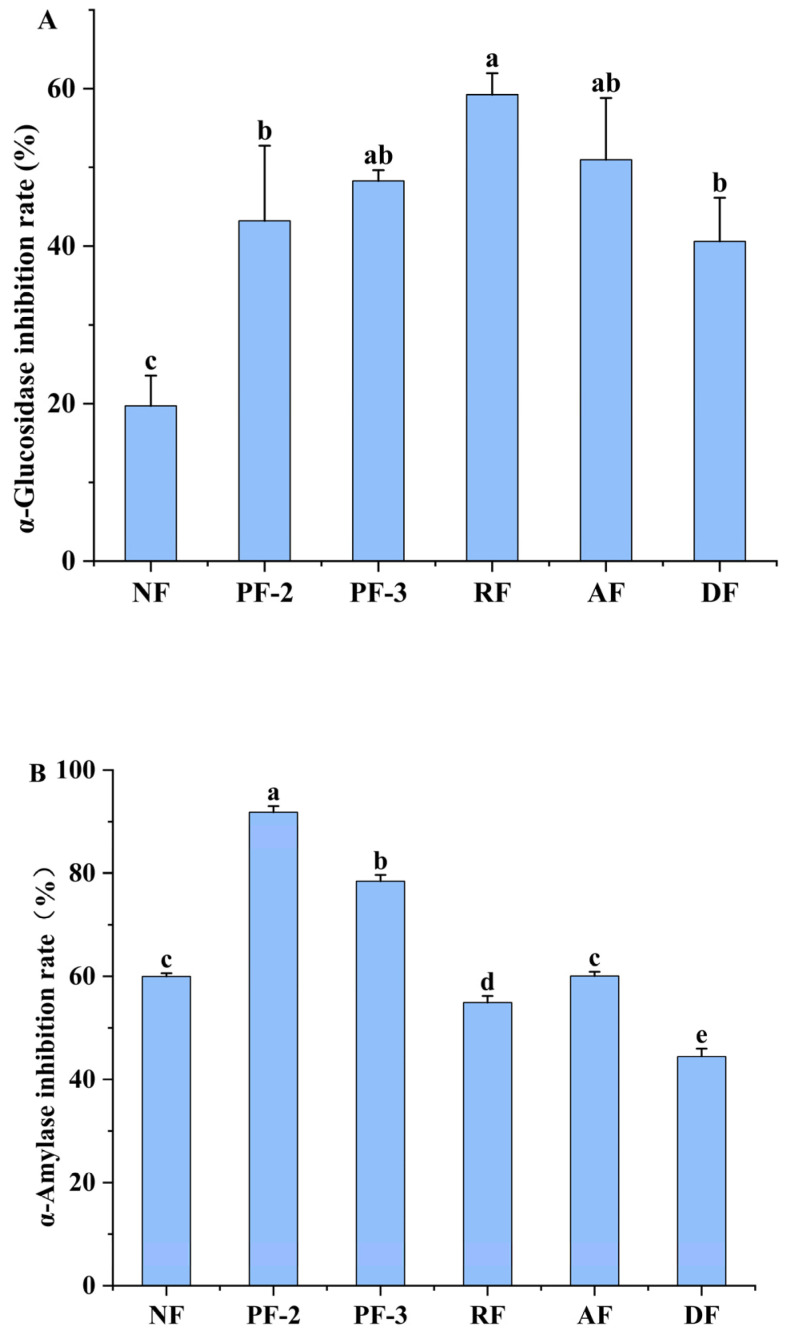
Inhibition of α-glucosidase (**A**) and α-amylase (**B**) activities in *Lactobacillus*-fermented *Sargassum fusiforme*. NF, non-fermented supernatant of *S. fusiforme*; PF-2, *L. plantarum* FY02-fermented supernatant of *S. fusiforme*; PF-3, *L. plantarum* FY03-fermented supernatant of *S. fusiforme*; RF, *L. rhamnosus* GJ01 fermented supernatant of *S. fusiforme*; AF, *L. acidophilus*-fermented supernatant of *S. fusiforme*; and DF, *L. delbrueckii*-fermented supernatant of *S. fusiforme*. Values are presented as the means ± standard deviation (n = 3). Different lowercase letters above bars denote significant differences between treatments (*p* < 0.05).

In line with expectations, compared with the non-fermented *S. fusiforme*, we detected marked 2.0- to 3.0-fold increases in the inhibition of α-glucosidase activity in *S. fusiforme* fermentation products, with the most pronounced effect (a 59.23% ± 3.91% inhibition) being detected in response to fermentation with *L. rhamnosus* (Figure 7A).

α-Amylase hydrolyzes polysaccharides (e.g., starch), yielding oligosaccharides that are subsequently converted into monosaccharides by enzymes such as maltase and sucrase in the small intestine, whose absorbance results in a rapid elevation in blood glucose levels [44]. Consequently, the inhibition of α-amylase activity can contribute to delaying the onset of hyperglycemic symptoms. As shown in Figure 7B, among the assessed fermentation treatments, we found that compared with the non-fermentation treatment, there was a 91.83% ± 1.19% inhibition of the α-amylase activity of *S. fusiforme* fermented with *L. plantarum* FY02, representing a 53.10% increase. Similarly, in response to fermentation with *L. plantarum* FY03, we detected a 78.45% ± 1.23% inhibition of α-amylase, representing a 30.79% increase compared with that measured in the non-fermentation treatment. However, not all of the assessed strains promoted a comparable increase in the rate of α-amylase inhibition. For example, compared with non-fermentation, we detected only a small difference in the rates of α-amylase inhibition when fermenting with *L. acidophilus* (*p* > 0.05), and, indeed, there were significant reductions in the inhibition of α-amylase activity when fermenting with *L. rhamnosus* and *L. delbrueckii* (*p* < 0.05). We accordingly speculate that these differences may reflect the different capacities of different LAB strains to degrade active substances that inhibit digestive enzymes, such as α-amylase.

In light of the absence of reports regarding the lactobacilli fermentation of *S. fusiforme* and its practical production, this study was conducted solely on a laboratory scale to examine the influence of lactobacilli fermentation on *S. fusiforme* and to elucidate the fermentation parameters under controlled conditions, thereby providing guidance for future production endeavors. Nonetheless, if particular industrialized production parameters are sought, research on large-scale production is essential, considering the disparities between the actual production conditions and laboratory settings.

In summary, the fermentation of *S. fusiforme* using lactic acid bacteria proposes a novel approach to valorize traditional food and algal resources, which establishes a theoretical foundation for manufacturing ready-to-eat fermented algal products, such as tablets and beverages. These studies analyzed the bioactive compounds in the supernatant of *S. fusiforme* before and after fermentation to evaluate the beneficial changes mediated by Lactobacillus fermentation. These findings provide a theoretical foundation for scaling up the industrial production of *S. fusiforme* through Lactobacillus fermentation, aiming to enhance its functional properties and diversify product formats. These studies provide valuable insights for advancing functional food development. However, further purification, characterization, and analysis are necessary to advance pharmaceutical development and enable the clinical translation of these findings. Given the complex composition of the processed *S. fusiforme* supernatant as a food matrix, this study utilized in vitro experiments. Specifically, we selected validated antioxidant (e.g., DPPH/ABTS radical scavenging) and hypoglycemic activity assays (e.g., α-glucosidase inhibition), which are well established in previous studies to evaluate functional properties [21,22,23,24,25]. While in vitro techniques are highly reproducible and controllable under laboratory conditions, accurately replicating the complexities of in vivo physiological systems remains a major technical hurdle [45]. The physicochemical properties of *S. fusiforme* exhibited significant changes following fermentation, as evidenced by in vitro experiments that highlighted the potential functional impact of these compositional alterations. Nevertheless, results obtained through in vitro approaches must be interpreted with caution when extrapolating to in vivo outcomes. The in vitro model employed here is insufficient to support definitive conclusions regarding the antioxidant and hypoglycemic effects of Lactobacillus-fermented *S. fusiforme* products, primarily because in vitro systems cannot fully replicate the complexity of in vivo physiological environments.

## 4. Conclusions

Our findings in this study revealed that the *Lactobacillus*-mediated fermentation of *S. fusiforme* can contribute to reductions in the pH and increases in total acid concentrations of the culture supernatants, thereby indicating that these bacteria can utilize *S. fusiforme* as an energy source. However, not all of the assessed strains promoted significant enhancements in the contents of polyphenols and total flavonoids, antioxidant activities, or the inhibition of α-glucosidase and α-amylase activities during fermentation. These findings thus indicate differences in the capacities of different *Lactobacillus* strains to degrade *S. fusiforme*, and that there may also be potential synergistic interactions between different strains with respect to metabolism of the active constituents in *S. fusiforme*. In addition, we detected significant increases in SCFA contents during fermentation, which could have beneficial effects regarding intestinal health. Collectively, these findings highlight the potential utility of *S. fusiforme* as a fermented food substrate, with fermentation serving as an efficient processing technique that contributes to augmenting flavor and elevating nutritional value.

In summary, our findings in this study provide evidence to indicate that *S. fusiforme* can function as a novel fermentation substrate. The fermentation of *S. fusiforme* with strains of *Lactobacillus* was demonstrated to be effective in enhancing the nutritional value of this seaweed, conferring improved antioxidant and hypoglycemic activities. However, this study was based exclusively on in vitro experiments, thereby highlighting the need for further animal studies to verify these enhancements and to assess the safety of fermented *S. fusiforme*.

## Figures and Tables

**Table 1:** Contents of short-chain fatty acids (μg/mL) in the supernatant of fermented *Sargassum fusiforme*.

	NF	PF-2	PF-3	RF	AF	DF
Acetic acid	14.50 ± 1.97 ^e^	137.26 ± 12.31 ^c^	117.16 ± 3.05 ^d^	173.54 ± 0.32 ^b^	185.76 ± 6.71 ^b^	403.04 ± 3.38 ^a^
Isohexanoic acid	4.11 ± 0.01 ^a^	3.08 ± 0.47 ^b^	4.14 ± 0.46 ^a^	4.00 ± 0.49 ^ab^	3.20 ± 0.30 ^ab^	4.02 ± 0.21 ^ab^
Propanoic acid	0.37 ± 0.02 ^c^	0.50 ± 0.07 ^bc^	0.51 ± 0.03 ^bc^	0.74 ± 0.02 ^a^	0.54 ± 0.11 ^b^	0.80 ± 0.01 ^a^
Isobutyric acid	0.56 ± 0.06 ^a^	0.60 ± 0.13 ^a^	0.57 ± 0.01 ^a^	0.72 ± 0.10 ^a^	0.73 ± 0.20 ^a^	0.59 ± 0.03 ^a^
Butanoic acid	0.11 ± 0.02 ^d^	0.09 ± 0.00 ^d^	0.10 ± 0.00 ^d^	0.22 ± 0.01 ^b^	0.15 ± 0.01 ^c^	0.26 ± 0.01 ^a^
Isovaleric acid	0.19 ± 0.03 ^d^	0.24 ± 0.04 ^cd^	0.22 ± 0.00 ^cd^	0.55 ± 0.00 ^a^	0.27 ± 0.04 ^bc^	0.32 ± 0.01 ^b^
Valeric acid	0.05 ± 0.00 ^a^	0.03 ± 0.02 ^a^	0.03 ± 0.01 ^a^	0.08 ± 0.00 ^a^	0.05 ± 0.02 ^a^	0.08 ± 0.07 ^a^
Hexanoic acid	0.20 ± 0.01 ^a^	0.16 ± 0.00 ^bc^	0.16 ± 0.01 ^bc^	0.16 ± 0.00 ^b^	0.16 ± 0.00 ^bc^	0.15 ± 0.00 ^c^

Values denoted by the same lowercase letters indicate an absence of substantial variation between treatments, whilst different letters indicate significant differences (*p* < 0.05).

**Table 2:** Qualitative analysis of volatile substances in *Sargassum fusiforme*.

Count	Class	Name	CAS	Odor Description
1	Ketones	Cyclopentanone	120-92-3	Minty
2		Cyclohexanone	108-94-1	Minty acetone
3		2-Butanone, 3-hydroxy	513-86-0	Sweet, buttery, creamy
4		3-Pentanone	96-22-0	Ethereal acetone
5		2-Butanone	78-93-3	Ethereal, fruity
6		1-Penten-3-one	1629-58-9	Pungent, peppery
7		4-Methyl-2-pentanone	108-10-1	Fruity
8		2-heptanone	110-43-0	Fruity, spicy
9	Alcohols	3-Methylbutanol	123-51-3	Slight smell of alcohol
10		1-Pentanol	71-41-0	Pungent, bready
11		2-Methyl-1-propanol	78-83-1	Ethereal, winey
12		1-Butanol	71-36-3	Strong smell of alcohol
13		3-Methyl-2-butanol	598-75-4	Fruity
14		4-Methyl-2-pentanol	108-11-2	Pungent alcohol
15		2-Methyl-2-propanol	75-65-0	Camphor
16		1-Propanol	71-23-8	Alcoholic, fermented
17		Propanediol	57-55-6	Odorless with a very slight alcoholic aroma
18		3-Methyl butanol	123-51-3	Alcoholic, pungent, ethereal, fruity
19		1-Hexanol	111-27-3	Pungent, fruity
20	Aldehydes	1-Octanal	124-13-0	Fruity
21		1-Nonanal	124-19-6	Waxy, aldehydic, citrus,
22		Propanal	123-38-6	Pungent
23		2-Methyl-2-propenal	78-85-3	Floral
24		Heptaldehyde	111-71-7	Fresh
25		3-Methylbutanal	590-86-3	Ethereal, chocolate, peach
26		(E)-2-nonenal	18829-56-6	Fats, cucumber, aldehydes, citrus
27		(E)-2-Heptenal	18829-55-5	Fruity overtones
28		*n*-Pentanal	110-62-3	Fermented, bready
29	Esters	2-methylpropyl 2-methylpropanoate	97-85-8	Ethereal, chocolate
30		Acetic acid ethyl ester	141-78-6	Fruity—grape, sweet, rum-like
31		Pentyl acetate	628-63-7	Fruity
32		Ethyl (E)-2-butenoate	623-70-1	Pungent, chemical, diffusive
33		Isovaleric acid, methyl ester	556-24-1	Apple, fruity
34	Amines	Dibutylamine	111-92-2	Weak smell of ammonia
35	Olenes	Myrcene	123-35-3	Anise, grape, fruity
36	Acids	Acetic acid	64-19-7	Sour vinegar

Odor descriptions of the volatile compounds were referenced using the Perflavory Information System.

## Data Availability

The original contributions presented in the study are included in the article, further inquiries can be directed to the corresponding author.
